# Supplementary material for: Insights into the formation and evolution of extraterrestrial amino acids from the asteroid Ryugu
Source: Nat Commun. 2023 Mar 17;14:1482. doi: 10.1038/s41467-023-37107-6 (PMC10023693; doi:10.1038/s41467-023-37107-6)
Supplement: Supplementary file 1 — Supplementary Information [file 41467_2023_37107_MOESM1_ESM.pdf]

Supplementary information for "Insights into the formation and evolution of extraterrestrial amino acids from the asteroid Ryugu"

Christian Potiszil[1]*, Tsutomu Ota[1], Masahiro Yamanaka[1], Chie Sakaguchi[1], Katsura Kobayashi[1], Ryoji Tanaka[1], Tak Kunihiro[1], Hiroshi Kitagawa[1], Masanao Abe[2,3], Akiko Miyazaki[2], Aiko Nakato[2], Satoru Nakazawa[2], Masahiro Nishimura[2], Tatsuaki Okada[2,4], Takanao Saiki[2], Satoshi Tanaka[2,3,5], Fuyuto Terui[2,6], Yuichi Tsuda[2,3], Tomohiro Usui[2], Sei-ichiro Watanabe[7], Toru Yada[2], Kasumi Yogata[2], Makoto Yoshikawa[2,3] and Eizo Nakamura[1]

[1]Pheasant Memorial Laboratory, Institute for Planetary Materials, Okayama University, Yamada 827, Misasa, Tottori, 682-0193, Japan.

[2]Institute of Space and Astronautical Science, Japan Aerospace Exploration Agency, Sagamihara, Japan.

[3]The Graduate University for Advanced Studies (SOKENDAI), Hayama, Japan.

[4]University of Tokyo, Tokyo, Japan.

[5]University of Tokyo, Kashiwa, Japan.

[6]Kanagawa Institute of Technology, Atsugi, Japan.

[7]Nagoya University, Nagoya, Japan.

*Corresponding author - Email: cpotiszil@okayama-u.ac.jp

Validation and Quantitation

Three calibration curves were made for each amino acid and the limit of detection (LOD) and limit of quantitation (LOQ) calculated by 3x and 10x the standard deviation of the y-intercept divided by the slope of the calibration curve, respectively. The concentration of the Ryugu amino acids was then determined using the peak area obtained from the extracted ion chromatograms for each amino acid and for each of the three runs of the sample. An average was calculated from the 3 values obtained for a given amino acid and blank corrected by subtracting the average peak area value of the blank. The blank corrected average peak area value was divided by the most similar peak area value from the standard and then multiplied by the concentration of that amino acid in the standard. Finally, the Ryugu amino acid concentrations were compared to the LOD and LOQ to establish if they were above the LOD and LOQ.

Tandem Mass Spectrometry

Further to the identification of amino acids using the retention time of the amino acids in extracted ion chromatograms (EIC) from standards and the Ryugu particles, some amino acids were present in high enough concentrations to obtain tandem MS (mass spectrometry) data (MS^2) (Extended Data Figure 1-3). The MS^2 records the fragment ions formed when the parent ion of the amino acid is collided with by a N_2 molecule. The MS^2 data are more specific to particular compounds and their isomers. For example, glycine and β-alanine record distinct MS^2 patterns. While the use of retention

time is sufficient, the MS[2] data provides additional evidence to support the correct identification of the amino acids reported here.

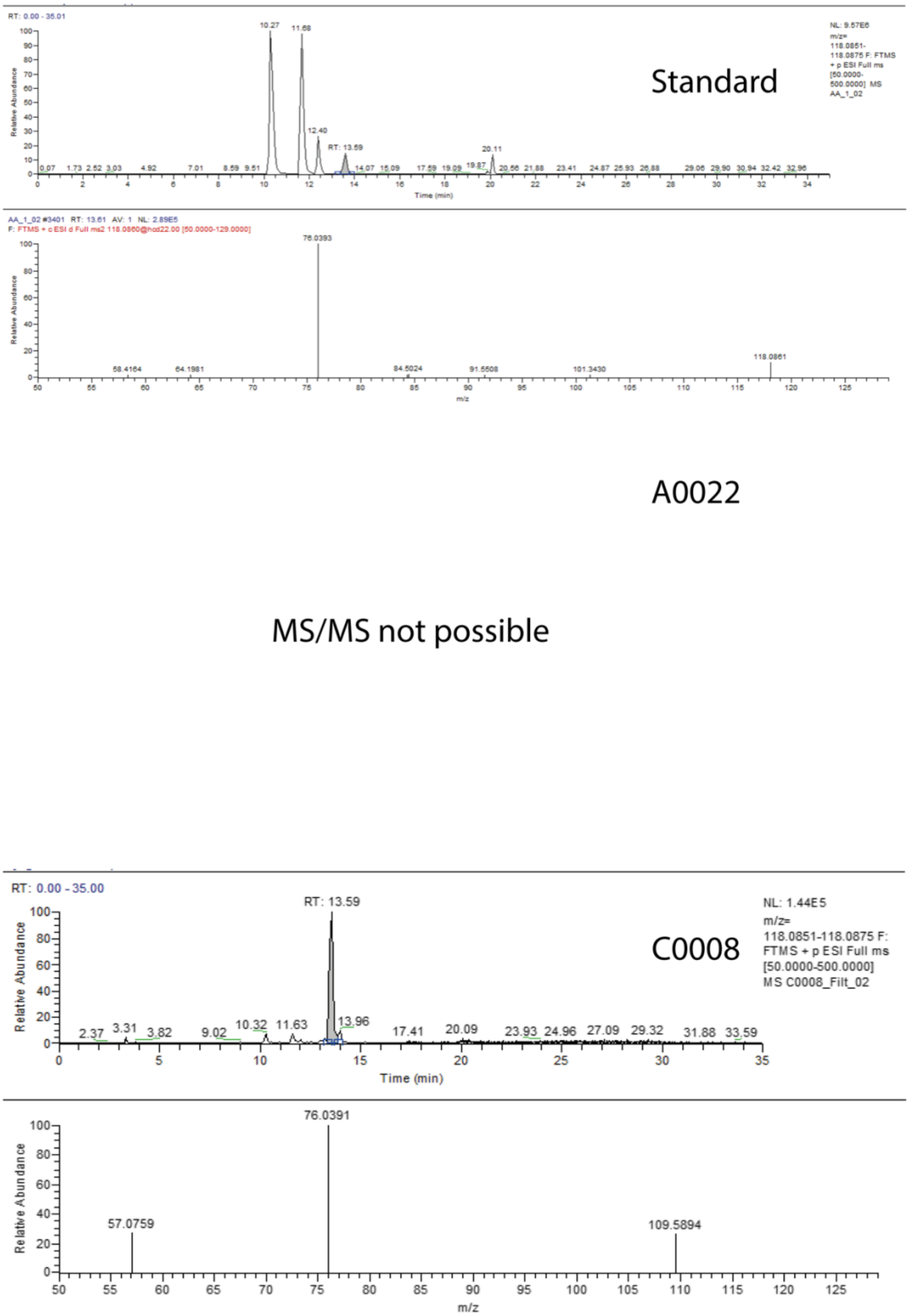

Supplementary Figure 1: Tandem MS (MS^2) data for glycine in a 1 µg/g standard and the Ryugu particles. Note that the measured intensity of glycine for the A0022 sample was not high enough to obtain MS^2 data. The MS^2 pattern resulting from colliding the analyte with N_2 gas is the same for the

standard and C0008, indicating that the analyte in C0008 is indeed glycine as an isopropyl ester. Note that the parent ion peak is missing in the pattern for C0008, likely due to the low concentration of the analyte compared to the standard.

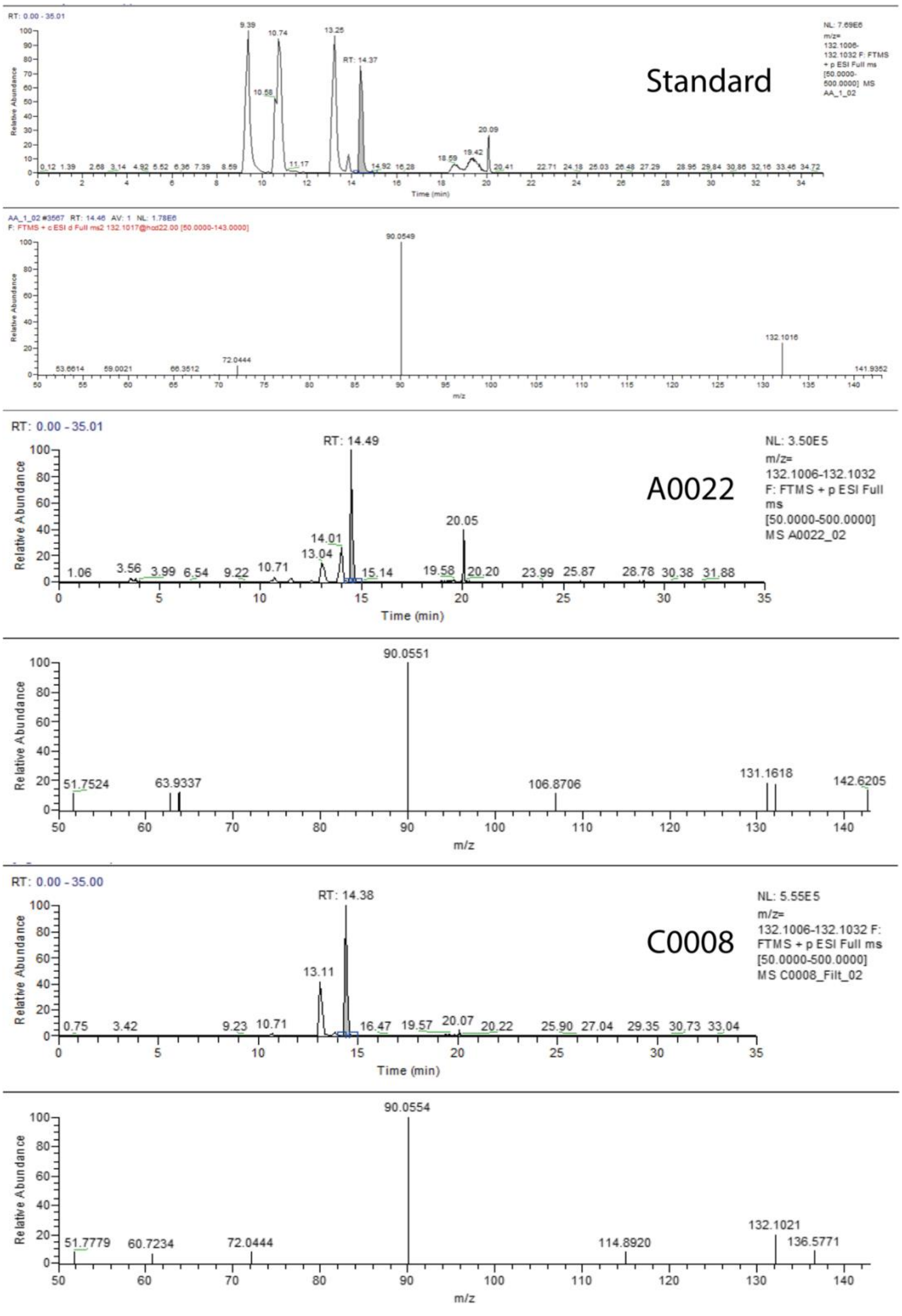

Supplementary Figure 2: Tandem MS (MS^2) data for β-alanine in a 1 µg/g standard and the Ryugu particles. The MS^2 pattern resulting from colliding the analyte with N_2 gas is the same for the

standard and A0022 and C0008, indicating that the analyte in the Ryugu particles is indeed β-alanine as an isopropyl ester.

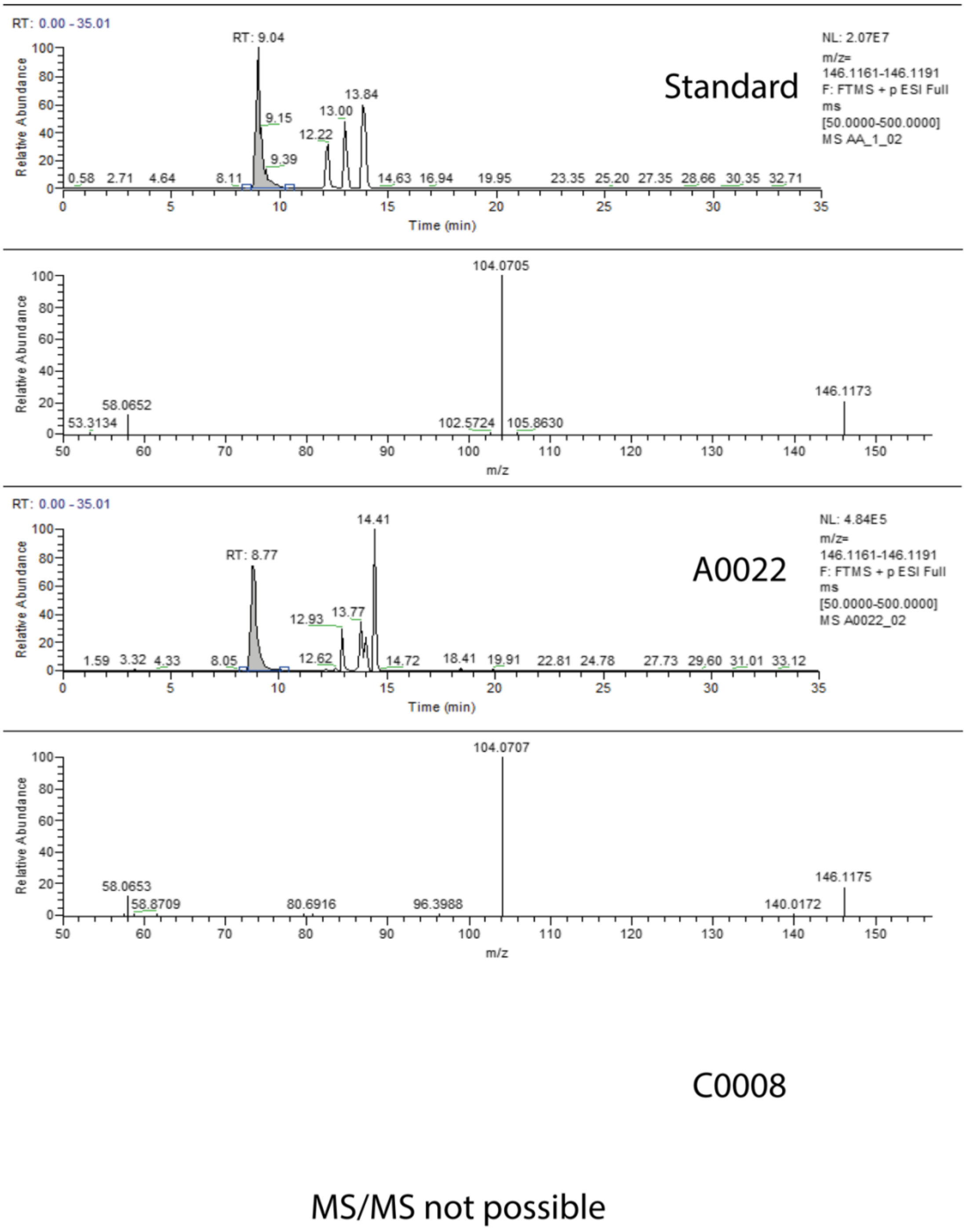

Supplementary Figure 3: Tandem MS (MS^2) data for N,N-dimethylglycine in a 1 µg/g standard and the Ryugu particles. Note that the measured intensity of glycine for the C0008 sample was not high enough to obtain MS^2 data. The MS^2 pattern resulting from colliding the analyte with N_2 gas is the

same for the standard and A0022, indicating that the analyte in A0022 is indeed N,N-dimethylglycine as an isopropyl ester.
